# Gain and loss: Human and environmental wellbeing – drivers of Kilimanjaro’s decreasing biodiversity

**DOI:** 10.1371/journal.pone.0334184

**Published:** 2025-10-29

**Authors:** Andreas Hemp, Mieko Miyazawa, Pekka Hurskainen

**Affiliations:** 1 Department of Plant Systematics, University of Bayreuth, Bayreuth, Germany; 2 Waldkundeinstitut Eberswalde, Eberswalde, Germany; 3 Global Mountain Biodiversity Assessment, University of Bern, Bern, Switzerland; 4 Department of International Studies, Graduate School of Frontier Sciences, The University of Tokyo, Kashiwa, Chiba, Japan; 5 Finnish Environment Institute, Helsinki, Finland; 6 Department of Geosciences and Geography, Earth Change Observation Laboratory, University of Helsinki, Helsinki, Finland; University of Ferrara, ITALY

## Abstract

Tropical mountains such as Kilimanjaro are biodiversity hotspots providing ecosystem services for millions of people, but many are under great pressure. Effective policies to halt biodiversity loss require an understanding of which anthropogenic factors are the main direct causes. While previous research focused mainly on climate change and on the effects rather than the causes, we investigated the effects of multiple drivers on biodiversity. The focus is on floristic and vegetation diversity, as vegetation is closely related to the diversity of other taxa and plays a fundamental role in ecosystem functioning. We show that land-use change caused by rapid population growth was the main direct driver on Kilimanjaro between 1911 and 2022, when 75% of natural species per km^2^ disappeared from the lower slopes. Climate change, on the other hand, had no apparent influence on the observed trends in biodiversity. The significant increase in traditional and diverse agroforestry and the establishment of protected areas show possibilities for mitigation. Kilimanjaro is thus an example of the challenges of global change, but also of the prospects and opportunities for other tropical regions.

## Introduction

Globally, human population growth and increasing per-capita demands for natural resources and energy are escalating human impacts on nature [1]. These indirect factors — such as economic development, institutions, and governance — contribute to biodiversity loss and influence direct anthropogenic drivers, which affect biodiversity at multiple levels (within species, between species, and across ecosystems) [2]. The most notable direct drivers include land-use change, climate change, natural resource exploitation, pollution, and invasive species [3]. Human well-being relies on the biodiversity of ecological systems and the benefits they provide to humans (known as nature’s contribution to people, NCP, formerly “ecosystem services”) [4–6], making anthropogenic impacts on nature a growing concern [7,8]. While climate change has attracted much attention as one of the drivers of biodiversity loss [8–10], it is not the only one [11–13], necessitating further examination of which drivers have the greatest effects on biodiversity [14]. Additionally, research has mainly focused on impacts rather than the drivers of global environmental change [15,16].

Kilimanjaro, the world’s highest free-standing mountain, features diverse ecosystems, high species diversity, and distinct land use systems [17]. The NCP provided by this tropical volcano includes climate and water regulation, food and timber provision and the spillover of crop pollination services into adjacent agro-ecosystems [18,19]. Due to increasing human pressures, Kilimanjaro’s ecosystems face severe threats [20,21], classifying it as a biodiversity hotspot [22]. It exhibits typical aspects of environmental change in tropical Africa while combining various features and challenges on one mountain, making it an excellent model for the investigation of the relationship between nature and humans.

The objective of this study is to investigate how the main direct and indirect anthropogenic factors (with the exception of pollution, for which no data are available) affect biodiversity at Kilimanjaro and to assess the role of conservation in relation to biodiversity. The focus is on floristic and vegetation diversity, as vegetation is closely related to the diversity of other taxa and plays a fundamental role in ecosystem functioning and thus NCP. Our biodiversity assessment is restricted to richness of plant species (overall species numbers and numbers of indicator species).

First, we investigate the spatial and temporal relationship between population growth and economic development (as indirect drivers) and land use (as a direct driver) by relating changes in population density and economic development to changes in land use and vegetation cover. For this, we use historical German maps and census data from the beginning of the 20th century to get an overview of the most important changes between 1911 and 1976. A more detailed insight into the situation since then is provided by the availability of satellite imagery. We focus on the inhabited areas of Kilimanjaro below the national park where no people live. Furthermore, we convert these changes in land cover into changes in biodiversity by using the number of species of different plant groups as an indicator. To do this, we use a large dataset of 1600 vegetation plots with almost 3000 plant species that have been established throughout the mountain range since 1996.

With this approach we want to answer the following questions:

(i) Which of the anthropogenic factors addressed have the greatest impact on biodiversity and how have the impacts of these anthropogenic factors changed in recent decades?
(ii) How does climate change affect biodiversity?
(iii) How is biodiversity changing?
(iv) Can nature conservation mitigate the negative effects?

We hypothesize the following:

(i) Population growth is the main indirect driver of biodiversity change, while land use is the main direct driver. Demographic change differs between rural and urban areas: The population in urban areas is growing faster than in rural areas, and the population is more stable in mountainous areas than in lowland areas. Therefore, land cover changes are more pronounced in the lowlands than in the highlands.
(ii) Climate change affects biodiversity mainly through changes in land cover after catastrophic events (e.g., fires after droughts or landslides after heavy rainfall), while the direct effects are minor.
(iii) Species diversity is generally decreasing, but differently for different plant groups.
(iv) Direct anthropogenic impacts inside the nature reserves have decreased, while they are increasing outside the reserves.

## Materials and methods

### Study area

**Fig 1:**
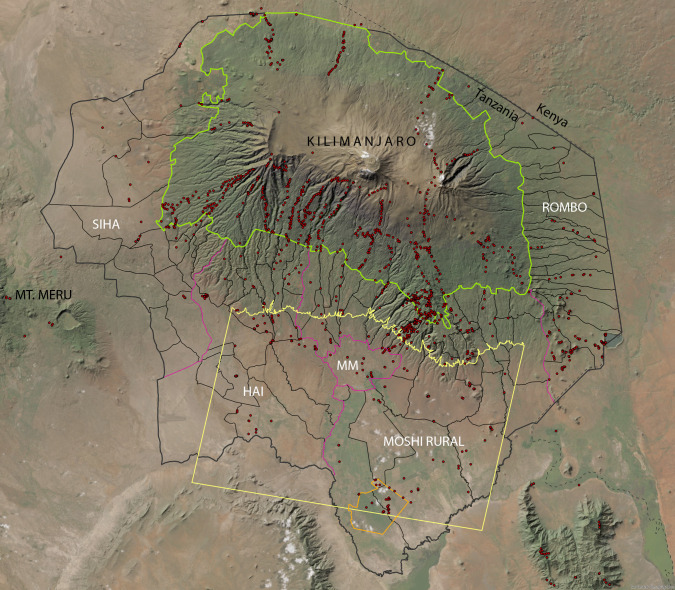
Study area. Study area (outer thick grey line) with delineation of the wards, situation of 2022 with 94 wards (grey lines), five districts (red lines; MM: Moshi Municipal), the border of KINAPA (green line), international border (dashed/dotted line), location of the map in Fig 10 (yellow line), Namalok nature reserve (orange line) and the available vegetation plots (red dots). Map source: Reprinted from World Imagery Basemap (Esri, Maxar, Earthstar Geographics, and the GIS User Community) under a CC BY license, with permission from Esri Deutschland GmbH, original copyright 2024.

Kilimanjaro, located in North-Eastern Tanzania, is a UNESCO World Heritage site, boasting approximately 3000 vascular plant species [23], representing about one-third of Tanzania’s approximately 10,000 vascular plant species [24]. The study area encompasses the inhabited lower slopes and foothills below Kilimanjaro National Park (KINAPA) with 1688 km^2^ within five districts of Kilimanjaro Region: Moshi Municipal, Moshi Rural, Hai, Rombo, and Siha, covering 3282 km^2^ (Fig 1).

**Fig 2:**
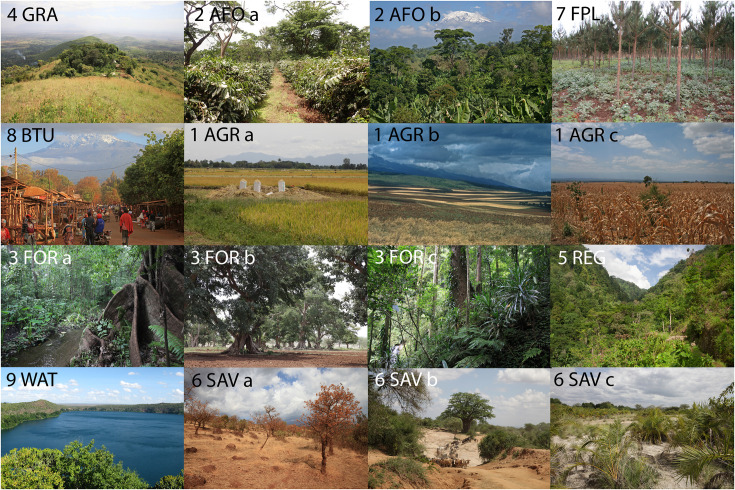
The nine land cover/land use (LULC) classes used for the study area. The first two lines represent anthropogenic LULC classes. GRA: submontane grassland (class 4); AFO: agroforestry (class 2), a: commercial coffee plantation, b: traditional agroforestry system of a Chagga homegarden; FPL: forest plantation (class 7); BTU: built-up area (class 8); AGR: agriculture (class 1), a: rice field, b: wheat field, c: maize field. The last two lines represent natural LULC classes. FOR: forests (class 3), a: groundwater forest, b: *Ficus* riverine gallery forest, c: submontane *Newtonia* ravine forest; REG: forest regeneration (class 5); WAT: waterbody of Lake Chala (class 9); SAV a and b: savanna grasslands and woodlands, c: palm shrubland on alkaline soil with high groundwater table (class 6).

Surrounding the mountain between 700 and 1100m is a dry and hot colline savanna zone. This zone is characterized by a heterogeneous mosaic of disturbed savanna vegetation, croplands, and built-up areas (Fig 2). The remaining natural vegetation faces significant pressure from human activities such as agricultural expansion and intensification, urbanization, firewood collection, brick manufacturing, and grazing [21,25–27]. The predominant natural vegetation consists of savanna woodlands with species of the genera *Acacia, Terminalia, Combretum, Ozoroa* and *Sclerocarya* in the tree and shrub layer, but the woodlands have degraded typically into grasslands dominated by *Themeda, Eragrostis, Heteropogon, Chloris* and *Cenchrus* species. Remnants of tall forest canopy exist along the river courses with fig trees (mainly *Ficus sycomorus*), *Sorindeia, Trilepisium* and *Mimusops* and in some areas with high groundwater table closed albeit heavily disturbed lowland forests still exist. Here, *Oxystigma msoo* a near-endemic trees species occurs together with *Khaya anthotheca, Trichilia emetica* and *Milicia excelsa*. Rushes and Sedges (*Typha domingensis, Cyperus papyrus* and *Schoenoplectus corymbosus*) dominate the few still existing swamps. Alkaline-resistant grasses (*Sporobolus spicatus* and *S. robustus*) and shrubs (*Suaeda monoica, Salvadora persica, Megalochlamys revoluta* and *Dobera loranthifolia*) characterize the quite widespread dry vegetation on alkaline soils, while table palms (*Phoenix reclinata* and *Hyphaene compressa*) dominate on alkaline soils with higher groundwater.

**Fig 3:**
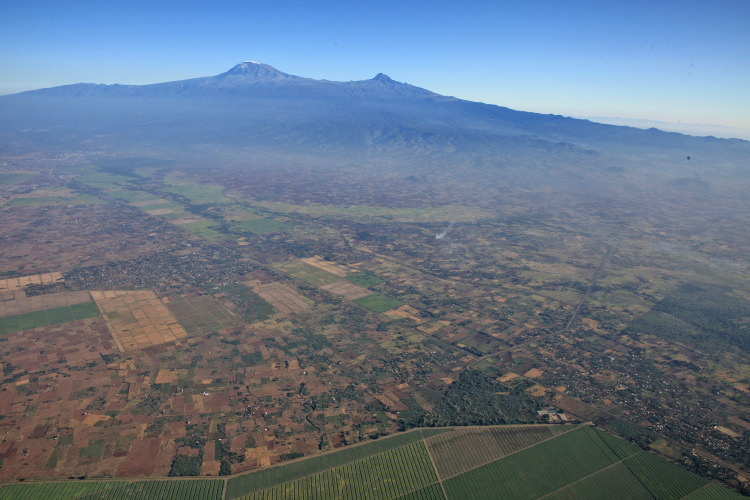
Cultivated lowlands on the southern slope of Kilimanjaro. In the foreground, the sugar cane plantations of TPC, surrounded by smallholder fields and – further up – rice fields, March 2025.

Smallholder farms cultivate extensive areas with maize, sunflower, beans, and millet. South of Moshi, large rice paddy fields and sugar cane plantations exist (Fig 3), while large wheat farms are present in the western part. During fallow periods, these fields are covered with ruderal vegetation consisting of mainly introduced species such as *Malvastrum coromandelianum*, *Acanthospermum hispidum, Bidens pilosa, Commelina benghalensis* and *Hyptis suaveolens*. A detailed physiographic map by Hemp et al. [28] illustrates the different land use and land cover types (LULC) of Kilimanjaro including our study area.

In the highlands within the submontane zone below KINAPA only small remnants of forest remain in the deepest valleys, where *Newtonia buchananii, Entandrophragma excelsum* (the tallest tree species of Africa [29]) and *Garcinia tanzaniensis* (a critically endangered tree species) grow. Everywhere else, the forests have mostly been converted into Chagga homegardens, a unique form of agroforestry prevalent on the southern and southeastern slopes. These homegardens feature four vegetation layers: Under a sparse tree layer (mainly *Albizia schimperiana*) bananas are grown, under the bananas coffee trees and on the ground vegetatables, maximizing land use efficiency and biodiversity, with over 500 plant species (most of them forest species) and endemic arthropods [30,31].

Steep, dry slopes in the valleys of the submontane cultivation zone are covered by species-rich mown *Hyparrhenia* grasslands, consisting of *Hyparrhenia rufa, Satureia abyssinica*, *Crepis carbonaria* and *Microchloa kunthii.* A detailed description of these land use and vegetation types is presented by [17,23,31–33]).

According to the Köppen and Troll/Pfaffen climate classification system (in [34]), the study area experiences a seasonal dry tropical climate influenced by the Inter-Tropical Convergence Zone. Rainfall occurs bi-modally, with rainy seasons from March to May and November. Rainfall and temperature vary with altitude and exposure to the dominant winds from the Indian Ocean [23]. Mean annual temperatures range between 15 and 25°C , while annual precipitation ranges from 400 to 2800 mm. Southern and eastern slopes receive more rainfall due to wind exposure from the Indian Ocean compared to the northern and western slopes [23,35]).

### Climate data

For the temperature analysis of the past decades, we used data from the NOAA National Climatic Data Centre (NCDC) (https://www.ncdc.noaa.gov/). The temperature data are much more limited than the precipitation data, as only a few weather stations have collected long-term temperature data. A station with useful data was Kilimanjaro Airport in the southwestern corner of the study area at 850 m a.s.l. The average daily temperature was determined from 4 to 24 measurements/day. For precipitation data, we used the published data from Otte et al. [36]. We used a generalised least squares (GLS) regression model to test the statistical significance of the long-term trends.

### Data of population and economic growth

The scale and time frame of our study were determined by the available census and remote sensing data: The smallest spatial unit for which census data were available was the ward level, an administrative structure for several villages, one single town or portion of a bigger town (Fig 1). The first available Landsat image for Kilimanjaro was from 1976, and the first eligible national population census was from 1978. The other censuses were from 1988, 1998, 2012 and 2022. Due to these limitations (including the availability of cloud-free satellite imagery), we analysed the years 1976 (using the 1978 census data), 2002 and 2022. As corresponding basis for analysing the historical maps from 1911 we used the 1913 census by mission statistics [37]. We used this data to calculate population density (number of inhabitants per km^2^) for analyses. In addition to population, we applied house building activity as a proxy for economic growth, as no data on per capita income or other indicators of economic development, such as ownership of superior goods (e.g., the number of motor vehicles), were available for the entire period. We used a global satellite-derived built-up area statistic called GHS-BUILT-S from the Global Human Settlement Layer (GHSL) project led by the Joint Research Centre of the European Commission [38]. In parallel, we used our land cover classification to delineate built-up areas (Table 1). For visualisation of these trends we digitized groups of buildings using time series of Google Earth images and the four 1:50,000 Ordnance Survey topographic sheets from the early 1990s (Sheet No: Moshi 56/4, Ol Molog 42/3, Rombo 57/1, Himo 57/3).

**Table 1:** The Land Use and Land Cover classification schema (cp. Fig 2).

LULC/ecosystem type	Description
(1) Agriculture	Maize, sunflower and millet fields of smallholder farmers, commercial farms with wheat fields on the western and northern slopes, sugarcane plantations and rice fields south of Moshi
(2) Agroforestry	Agroforestry including traditional Chagga home gardens and commercial coffee plantations with a sparse tree layer of *Albizia schimperiana*, *Rauvolfia caffra* and *Cordia africana*
(3) Forest	Moist forests including riverine and groundwater forests (with *Ficus sycomorus, Oxystigma msoo* and *Khaya anthotheca*) and submontane ravine forests with *Newtonia buchananii*, *Entandrophragma excelsum* and *Mitragyna rubrostipulata* in the tree layer
(4) Grassland	Mosaic of anthropogenic grasslands with *Hyparrhenia rufa, Bulbostylis densa* and *Sporobolus africanus* and agricultural fields in the submontane and lower montane zone, especially on steep slopes along the major river valleys and near KINAPA
(5) Regeneration	Forest regeneration with vegetation recovering after previous disturbances, mainly shrubland with *Lantana camara, Carissa edulis* and *Ochna insculpta*
(6) Savanna	Savanna with grasslands (*Cyperus niveus, Themeda triandra* and *Heteropogon contortus*), woodlands and dry forests with *Acacia nilotica, Combretum molle* and *Sclerocarya birrea*
(7) Forest plantation	Forest plantations with exotic tree species (*Pinus patula* and *Cupressus lusitanica*) on the northwestern and northern slopes
(8) Built-UP	Continuous impervious surfaces with infrastructure (buildings, roads, pavements, yards)
(9) Water body	Lakes

### Land cover

We used the LULC maps for 1976 and 2000 of Hemp [20,23] based on Landsat MSS imagery from 1976, and Landsat ETM+ imagery from 2000. To update the 2000 map to correspond to the 2002 census year, we made adjustments by visually inspecting various very high resolution (VHR) satellite imagery available at Google Earth and ESRI World Imagery platforms, correcting boundaries of agriculture/savanna and other classes. Similarly, we updated the physiographic map by Hemp et al. [28] to reflect the situation as of 2022.

Due to the lower spatial, spectral, and radiometric resolution of the Multi Spectral Scanner (MSS) on Landsat 1 satellite compared to the more advanced ETM+ and operational land imager (OLI) sensors on later Landsat satellites, the number of LULC classes had to be reduced to match what was realistically achievable to map from the 1976 Landsat 1 image. This led to a very rough categorisation of the main land cover and ecosystem types (Table 1):

Since we could not delineate built-up areas on the Landsat image from 1976, we used the oldest available topographic maps 1:50.000 from 1982. At that time, only Moshi existed as an urban centre. For the years 2002 and 2022, we used VHR satellite images in Google Earth to delineate the urban centres with built-up areas. The LULC maps were prepared using ArcMap 10.8.2. For more specifications of mapping these LULC classes see Supplementary Information S1 Text.

To quantify the change flows, or transitions, between LULC classes and the three time steps (1976, 2002 and 2022), we used the functions in OpenLand R library [39]. We visualized the change flows with a Sankey diagram, which illustrates relative transitions between the classes for the three time steps, as well as the net and gross changes with a stacked barplot.

In order to obtain an overview of the main changes in the landscape since the first census on Kilimanjaro in 1913 [37], we also analysed historical maps from the beginning of the 20th century. The map of Hans Meyer’s Ostafrika-Expedition 1911 [40] at a scale of 1: 300,000 provides very detailed and botanically sound descriptions of the vegetation, especially in the savanna areas of Kilimanjaro. For final delineation of the LULC types (see also S1 Text) we used additional historical information from the map and vegetation description by Meyer (1890) [41] together with information in Volkens (1897) [42], Jaeger (1909) [43] and Klute (1912) [44].

### Biodiversity

We used a subset of the 54 land cover types identified by Hemp [23,29,32] for the entire mountain range based on 1,600 releves. We combined the occurring plant communities to fit into our proposed nine classes to create species lists (see Supplementary Information S1 Text for more details).

The species numbers resulting from the analysis of the releves are listed in Table 2. The status of endemics (species with a restricted occurrence) follows a list by Gereau, Missouri Botanical Garden (pers. comm./unpublished data). We considered species in the following IUCN categories as Red List species (reviewed at https://www.iucnredlist.org/): Critically Endangered, Endangered, Vulnerable, Near Threatened. The status of neophytes (introduced species) was checked in FTEA (1952–2012) and Kew Plants of the World Online (https://powo.science.kew.org/).

**Table 2:** Species numbers in the study area based on the 772 releves.

LULC/ ecosystem type	Number of releves	Species number	Number endemics	Number IUCN Red List species	Number neophytes
Agriculture*	136	700	2	1	100
Agroforestry	76	581	4	0	138
Forest	101	846	25	16	62
Grassland	163	509	6	0	46
Regeneration	24	300	0	0	15
Savanna	272	1059	9	10	43
Water body	0	0	0	0	0
All classes	772	2057	35	26	180

*Including classes forest plantation and built-up (see Supplementary Information S1 Text)

Using these figures in order to calculate the number of species per km^2^ and to compare it with the human population density per km^2^ required two further steps. We first had to take into account the fact that the number of species increases with area. This species-area relationship (SAR) is one of the few very well documented patterns of species richness [45,46]. This model is still the most commonly used and is considered the best for describing species-area calculations [47]. We used the calculated SAR for the 13 major land use and vegetation classes of Kilimanjaro of Hemp et al. [48] to scale up the species counts of the nine classes to 1 km^2^.

Second, these classes show a certain overlap of species. For example, many species of open agricultural land also occur in agroforestry systems. Based on the analysis of the releves, we calculated this overlap which varied between the different classes and depended on the number of classes within a given area, in our case a ward. In one ward with all nine classes, the reduction factor increased to 0.51, meaning that the species numbers of each class had to be halved (see S1 Table with the species numbers and reduction factors for all combinations). Using the same approach, we calculated the number of endemic species, IUCN Red List species and neophytes, from the 772 releves (Table 2). In a final step, we used these reduced species numbers to calculate the final species numbers per km^2^ by relating them to the proportion of each LULC in the different wards.

We used the detailed LULC classification by Hurskainen et al. [75] from the southern slope of Kilimanjaro to demonstrate the correlation between different LULC and species richness on a finer scale.

### Data analysis

We related changes in human population density at the ward level, to changes in land use and vegetation cover in the respective wards. We then analysed the effects of land cover changes on biodiversity using the number of species of vascular plants as an indicator. The total number of species and the number of different plant groups (natural vegetation plants, endemic and Red List species, neophytes) were used as indicators. To analyse trends in species numbers, we fitted linear functions using R^2^ as the measure of fit. The bivariate correlation between biodiversity data as response variable and population and land use data as explanatory variable was performed with Pearson’s correlation coefficient using SPSS 29.0.2.0.

For critical remarks on the methodology see Supplementary Information S1 Text.

### Permits, inclusivity in global research

We obtained the necessary permits to access the sites and conduct fieldwork from the Tanzania Commission for Science and Technology (COSTECH), Tanzania National Parks (TANAPA), and the Tanzania Wildlife Research Institute (TAWIRI).

Additional information regarding the ethical, cultural, and scientific considerations specific to inclusivity in global research is included in the Supporting Information (S1 Checklist).

The individuals in this manuscript have given written informed consent (as outlined in PLOS consent form) to publish these case details.

## Results

### Demography

**Fig 4:**
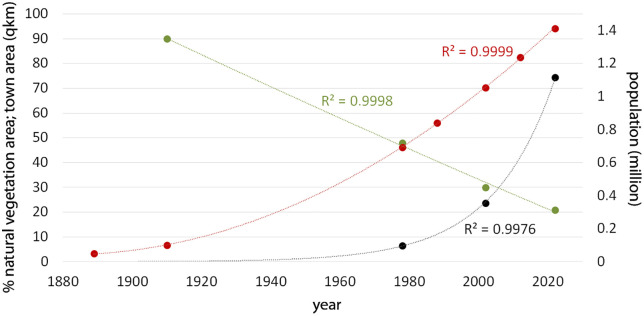
Human population growth, reduction of natural vegetation and increase of town area on Kilimanjaro. Human population growth during the last 133 years (red dots with polynomial regression of degree 2), starting in 1889 ([37,41], Bureau of Statistics 1968, 1998, 2003, 2013, 2022 [49]); reduction of natural vegetation (green dots with polynomial regression of degree 2) and increase of town area (black dots with exponential regression); correlation (Pearson’s r) of natural vegetation vs population (1911−2022) −0.985, p < 0.01 and vs town area (1976−2022) −0.915, ns.

The population around Kilimanjaro is experiencing rapid growth (Fig 4), rising from about 50,000 to over 1.4 million between 1889 and 2022 with an annual growth rate of 2.6%, and of 1.7% between 1976 and 2022 ([37,41], National Bureau of Statistics 1978, 2002, 2022 [49]).

### Climate

According to Otte et al (2016) consistently significant rainfall trends over the period 1973–2014 could not be found for the lowlands of Kilimanjaro. For the period 1974–2016 at the Kilimanjaro Airport there was a significant increase in mean temperature (GLS p < 0.001) with a mean annual increase of 0.027°C. Over this period there was no trend in maximum daily temperature, but a significant increase in daily minimum temperature (GLS p < 0.001) with a mean annual increase of 0.032°C.

### LULC changes

**Fig 5:**
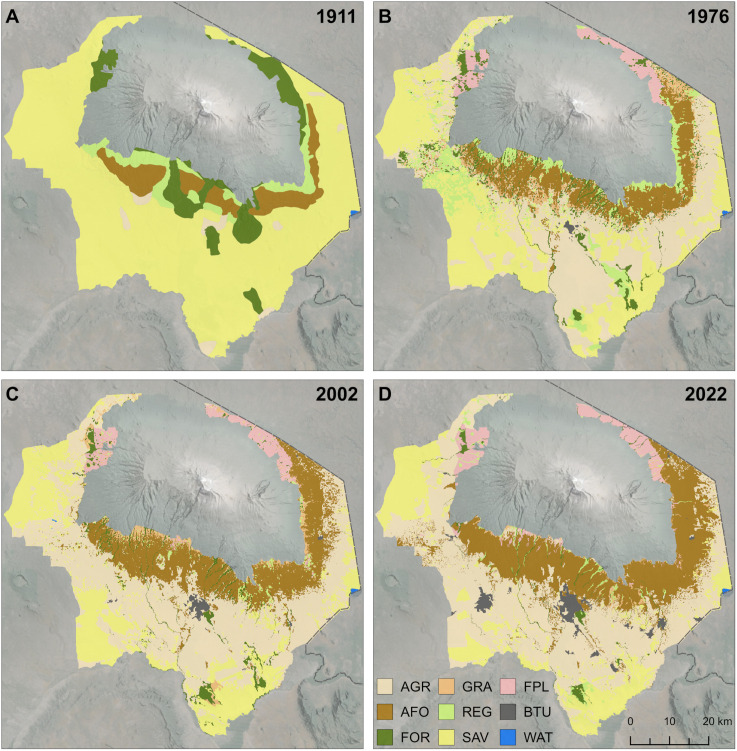
Maps of land cover/land use (LULC) on Kilimanjaro between 1911 and 2022. (A-D) LULC maps of 1911, 1976, 2002 and 2022. AGR. agriculture, AFO: agroforestry, BTU: built-up area, FPL: forest plantation, FOR: forest, GRA: anthropogenic grassland, REG: forest regeneration, SAV: savanna, WAT: water body. Reprinted from World Imagery Basemap (Esri, Maxar, Earthstar Geographics, and the GIS User Community) under a CC BY license, with permission from Esri Deutschland GmbH, original copyright 2024.

**Fig 6:**
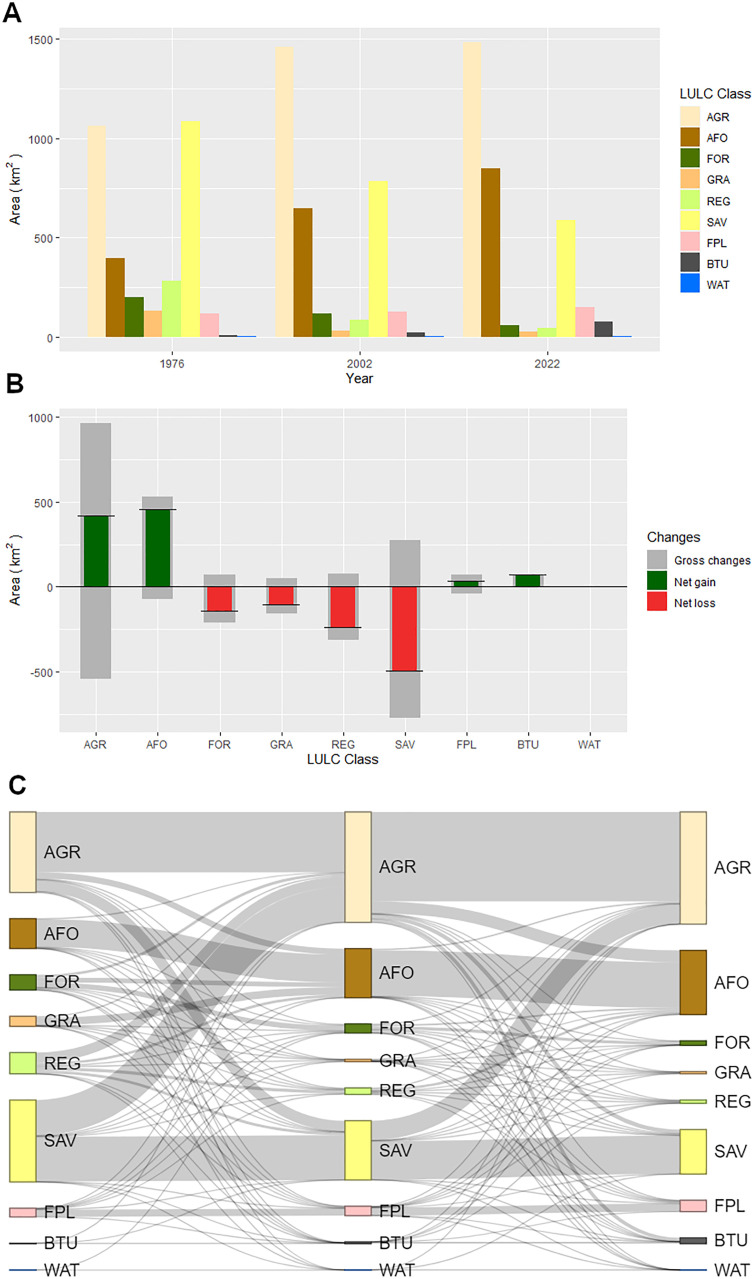
Changes of land cover/land use (LULC) on Kilimanjaro between 1976 and 2022. (A) Extent of LULC classes in 1976, 2002 and 2022. (B) Net and gross changes of LULC between 1976 and 2022. Anthropogenic LULC types increased on expense of natural vegetation. (C) Sankey diagram, illustrating relative LULC change flows between 1976 (left), 2002 (middle) and 2022 (right).

The spatial distribution of LULC classes for the four analysed time steps are shown in Fig 5A–D and Table 3, and the extent of each class for the period between 1976 and 2022 in Fig 6A. Areas of natural ecosystem types, including forest, regeneration, and savanna, decreased from 90% in 1911 to 19% in 2022 (Fig 4). In 1911 the extend of savanna was nearly at its natural maximum of 75%, with only 2% having been converted to agriculture in the lowlands. In 1976, the extent of savanna and agriculture were nearly identical, but agriculture then expanded to become the dominant LULC class.

**Table 3:** Land cover changes 1911-2022.

LULC/ ecosystem type	1911 area (km^2^)	1911 area (%)	1976 area (km^2^)	1976 area (%)	2002 area (km^2^)	2002 area (%)	2022 area (km^2^)	2022 area (%)
Agriculture	66	2	1062	32	1406	43	1486	45
Agroforestry	268	8	395	12	650	20	849	26
Forest	319 (500*)	10	201	6	119	4	60	2
Grassland	NA	NA	134	4	32	1	28	1
Regeneration	180	5	281	9	87	3	43	1
Savanna	2449	75	1090	33	795	24	588	18
Forest plantat.	0	0	116	4	170	5	150	5
Built-up	0	0	6	0	24	1	74	2
Water body	2	0	2	0	2	0	2	0

(*) estimated number including not covered riverine and ravine forests; NA: anthropogenic grasslands could not be delineated for 1911 (see Supplementary Information S1 Text).

Similarly, agroforestry expanded to 26%, surpassing savanna, which declined to 18%, becoming the second-largest land-use class in 2022. Apart from savanna, the other two natural vegetation types (forest and regeneration), also showed a steady decline. Forest plantations exhibited only minor changes in their extent, while built-up areas increased twelvefold between 1976 and 2022 (Fig 4).

The same patterns but from a different perspective can be seen in Fig 6B, which shows the net and gross changes across the period 1976–2022.

From this figure, two key insights arise: Agriculture and savanna were the most dynamic classes. Furthermore, all anthropogenic LULC classes experienced a net gain, while natural vegetation experienced a net loss across all categories.

The Sankey diagram (Fig 6C) provides further insights into LULC transitions by illustrating the relative changes between LULC classes across three time periods (1976, 2002, and 2022). The diagram highlights which LULC classes contributed to the expansion of specific categories. Notably, savanna conversion was the primary driver of agricultural expansion, with smaller contributions from other LULC classes.

### Biodiversity trends

The number of all plant species per km^2^ (mean at ward level) exhibited only a slight decrease of approximately 10%, between 1976 and 2022 (Table 4). The decline in plant species of natural habitats, including forest, regeneration, and savanna, was much more pronounced, with a decrease of 46%. Conversely, there was an increase of approximately 25% in neophytes during the same period. This means that not only is species richness changing, but also species composition, i.e., there is an increase in partially invasive and widespread species at the expense of native species with partially restricted distribution.

**Table 4:** Changes in species numbers 1976-2022 (mean at ward level).

	1976	2002	2022	Pearson’s r
All plants	704	640	636	−0.892 ns
Natural plants	340	250	184	−0.996*
Savanna plants	162	132	101	−1.000**
Neophytes	90	105	120	1.000**

Pearson correlation with population density: ** p < 0.01, * p < 0.05, ns: non significant.

## Discussion

### Demography and economic development as main drivers of land use changes

World population is projected to continue growing throughout the century, reaching between 9.6 billion and 12.3 billion at the end of the century, with Africa expected to experience at least a 3.5-fold increase in population. The current population of Africa, approximately 1.5 billion people in 2024, is projected to reach approximately 2.2 billion in 2054 [50]. Tanzania’s population growth rate stands at about 3.0% annually. If this trend persists, Tanzania’s population is estimated to increase from about 65 million in 2022 to approximately 95 million in 2050 and over 300 million by the end of the century [49,51].

The human population on Kilimanjaro, comprising Moshi Urban, Moshi Rural, Rombo, Siha, and Hai Districts, has multiplied 28 times over 133 years from about 50,000 in 1889 [41] and 100,000 in 1913 [37] to over 1.4 million in 2022 ([49], Fig 4). As a result, population density increased from 15 people/km^2^ in 1889–30 in 1913, 50 in 1948 [52], and 211 in 1978, eventually reaching 430 people/km^2^ in 2022. The local population density (outside the cities) rose to unprecedented levels of up to 1500 people/km^2^ in the most fertile highland area of the Chagga homegardens. This population growth resulted in a severe shortage of cultivable land and the subdivision of land into fragments too small to support a family and too numerous to allow for further expansion [25,53]). Consequently, many young people migrated from the highlands to urban centers such as Moshi, Arusha, and Dar es Salaam, or settled on the foothills of Kilimanjaro. This emigration from the highlands explains why the demographic growth rate on Kilimanjaro is lower than the national average in Tanzania and why the population growth rate in the lowlands (2.9%) was much higher than in the highland homegarden areas (0.9%) between 1976 and 2022. In 1976, approximately 30% of the population resided in the lowlands, a proportion that increased to about 50% by 2022.

**Fig 7:**
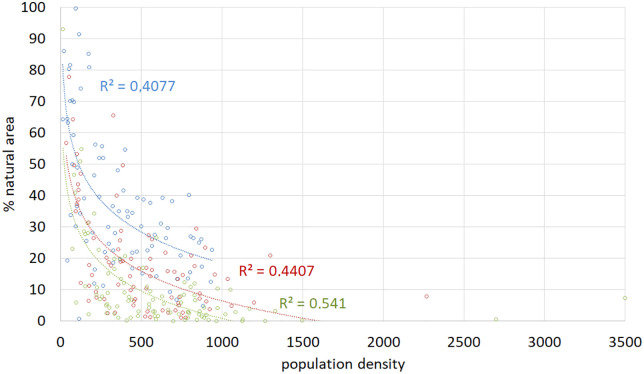
Changing share of natural habitats over time in correlation to population density per km^2^ at ward level on Kilimanjaro. Blue symbols 1976, red symbols 2002, green symbols 2022 with logarithmic regressions. Pearson’s correlation coefficient r 1976: −0.594, p < 0.001; 2002: −0.475, p < 0.001; 2022: −0.407, p < 0.001.

In the causal chain of factors affecting biodiversity, demography acts as an indirect driver via the direct drivers [7], at Kilimanjaro by controlling land use change as direct driver. In other regions of Tanzania, it was also the population growth that led to the expansion of cultivated land [54]. The observed growing population density results in the overall reduction of natural habitats since 1911 (Fig 4), with increasing annual reduction rates from 1.0%, 1.9% and 2.4% between 1911 and 1976, 2002 and 2022. This can be shown in more detail for the period 1976–2022 at ward level (Fig 7).

In addition to the clear correlation between increasing population density and decreasing natural vegetation, Fig 7 shows that not only the mean share of natural vegetation was higher in 1976 than in 2002 and 2022, as also seen in Fig 4, but also, that at a given population density, e.g., of 1000 people/km^2^, the natural vegetation share was approximately 18% in 1976, compared to 6% in 2002 and only 1% in 2022. This decline may be due to rapid economic development at an even higher rate than population growth increasing per capita demand for resources, e.g., via higher availability of machinery or pesticides, with this increasing land use intensity and reducing natural areas. Fig 3 gives an impression of the extent to which the vegetation at the southern foot of Kilimanjaro has been reshaped by humans.

For the entire Kilimanjaro region, including the five study districts and the Pare Mountains, Gross Domestic Product (GDP) grew about 5.7 times from 1980 to 2020 [49]. During this time, GDP per capita in Tanzania increased approximately 1.8 times [55], indicating that economic growth was much more rapid in Kilimanjaro. In our study area, reflected through construction activities as indicators of economic growth, Fig 4 shows an annual growth rate of built-up areas at 5.9% between 1976 and 2022, and 6.1% from 2002 to 2022, surpassing population growth. This aligns with global satellite-derived built-up area statistics named GHS-BUILT-S from the Global Human Settlement Layer (GHSL) project (see Supplementary Information S1 Text; Table 5). Trends post-2000 indicate significant expansion of built-up areas compared to the 1975–2000 period.

**Table 5:** Built-up surface (km^2^) from GHS-BUILT-S.

District	1975	2000	2020	1975 → 2000	2000 → 2020
Siha	3.34	4.97	10.70	+ 49%	+ 115%
Hai	7.82	12.08	20.85	+ 54%	+ 73%
Moshi Rural	23.26	32.02	42.93	+ 38%	+ 34%
Rombo	19.79	21.85	25.03	+ 10%	+ 15%
Moshi Municipal	6.32	9.82	11.04	+ 55%	+ 12%

This is also in line with trends on a continental level: Africa’s urban areas will more than double until 2025. The growth in urban area expansion is projected to outpace urban population growth, at 3.2% compared to 2.3% per year. The total urban footprint is projected to increase from 175 000 square kilometres to 450 000 square kilometres between 2020 and 2050 with an increasing number of urban agglomerations from 9,000 to over 11,000 [56].

**Fig 8:**
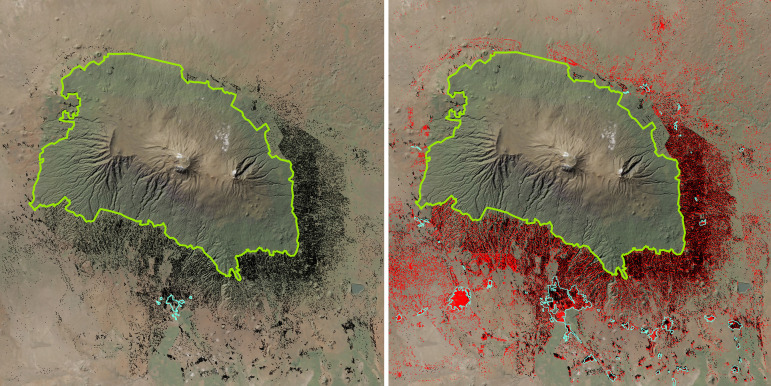
House building activities on Kilimanjao between 1990 and 2016. Left: Buildings (black dots) 1990; Right: New buildings (red dots) 2016. Blue lines: town centres; green line: KINAPA (National Park Boundary). Buildings digitized by Ethan Oleson based on time-series Google Earth images and the 1:50,000 Ordnance Survey topographic sheets. Reprinted from World Imagery Basemap (Esri, Maxar, Earthstar Geographics, and the GIS User Community) under a CC BY license, with permission from Esri Deutschland GmbH, original copyright 2024.

The trend of house building activities and the development of new town centers between 1990 and 2016, particularly in the lowlands of Hai and Siha districts on the southwestern side of Kilimanjaro, is illustrated in Fig 8. This aligns with GHS-BUILT-S (Table 5). The role of KINAPA as a barrier against encroachment by settlements is notable in Fig 8, highlighting the importance of governance through governmental protection of nature that delivers ecosystem services to Tanzania’s people.

Demographic and socio-economic factors have driven many land-use changes at Kilimanjaro. By 2000, a corridor of submontane forest that once connected Kilimanjaro and Mt. Meru had been largely replaced by human settlements and agriculture (Fig 5B). This forest corridor was crucial for the dispersal of forest animals [21], but Kilimanjaro is now increasingly isolated, impacting biodiversity and endemism.

On a positive note, the growth of Chagga homegardens – a sustainable land-use practice supporting various natural species [31,53,57] – has helped mitigate biodiversity loss, especially as these gardens have expanded at the expense of agricultural fields (Fig 5C). From 1976 to 2022, the area of homegardens more than doubled to 849 km^2^.

### Climate change was not an important driver of the observed biodiversity trends

A reanalysis of meteorological data for East Africa shows an approximate surface temperature increase of 0.95 K over the past 60 years (specifically between 2.5° to 5.0° South and 37.5° to 40.0° East). Additionally, the RCP6.0 scenario projects a further increase of 3 K by 2100 [58]. However, such modeled climate data for tropical mountains is sometimes very unreliable [35]. Beside increasing trends of temperature no consistently significant rainfall trends over the period 1973–2014 could be found for the lowlands of Kilimanjaro [36]. Consequently, we conclude that climate change did not significantly influence observed biodiversity trends or land use in the lowlands. If it had been a primary driver of land use change, we would expect to see negative impacts on subsistence agriculture and agroforestry, yet these categories showed significant gains. However, this differs from the situation above the cultivation belt within the national park. There, signs such as glacier retreat [59] indicate a gradual decline in regional humidity and precipitation, and an interplay between increasing human activities and decreasing humidity leads to an increasing frequency and intensity of forest fires, affecting biodiversity especially in the upper montane forest zone and the subalpine ericaceous belt [20]. Forest fires are also frequent in the humid lower montane forest zone near cultivated areas in the vicinity of larger anthropogenic clearings due to a changing microclimate with increasing air temperature and decreasing humidity [60]. In the last 120 years, Kilimanjaro has lost about 50% of its forest area [23]. This not only affects biodiversity, but also has far-reaching consequences for the regional climate. Deforestation leads to a significant increase in maximum air temperature and cloud base height [61], which on Kilimanjaro primarily affects the upper regions within the national park and even the glaciers [62] and impairs the area’s ability to provide vital NCPs, especially water. This interaction demonstrates that climate change is not always a challenge originating from outside but also a “homemade” problem caused by regional land-use change and boosting together biodiversity loss. However, such changes (e.g., the increase in wild fires) have not been observed in the lowlands of Kilimanjaro.

### Land use change: Main driver of biodiversity loss

Land use change and intensification are the main drivers of the decline of biodiversity and ecosystem services globally [3,5,63,64], exceeding the impact of climate change, particularly in tropical areas [11,14,65–67].

**Fig 9:**
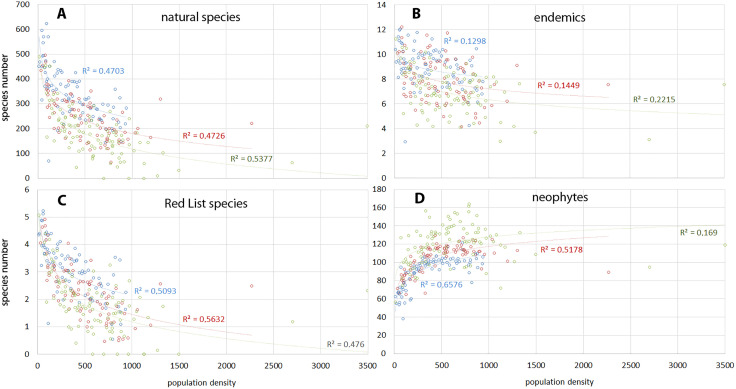
Biodiversity trends 1976-2022. (A) natural species, (B) endemics, (C) IUCN Red List species, (C) neophytes. Blue symbols 1976, red symbols 2002, green symbols 2022. Species numbers per km^2^ in correlation to population density at ward level with logarithmic regressions. Correlation indices are presented in Table 6.

For Kilimanjaro, Fig 9 illustrates the effects of population-induced natural habitat loss on species diversity. Species density of **natural species** (including endemics and Red List species) correlates closely with Fig 7, indicating that land-use change, notably ecosystem loss, is the primary cause of reduced species density (Fig 9A).

**Table 6:** Pearson correlation coefficient for densities of plant groups vs population (Fig 9).

	Natural	Endemic	Redlist	Neophytes
1976 (n = 82)	−0.673***	−0.399***	−0.717***	0.696***
2002 (n = 82)	−0.527***	−0.315**	−0.589***	0.459***
2022 (n = 94)	−0.488***	−0.341***	−0.419***	0.137 ns

n: number of wards, which changed after the division of several wards after 2002 (see Supplementary Information S1 Text). ***p < 0.001, **p < 0.01, ns: non significant.

The loss of about 40% of natural savanna habitats (76% since 1911) significantly threatens Kilimanjaro’s biodiversity, as savanna ecosystems are crucial for plant species diversity [17,68]. This decline also affects traditional medicinal plants, primarily found in Kilimanjaro’s lowlands [69]. This trend aligns with global patterns; over the last century, tropical savanna ecosystems have decreased by over 50% due to agricultural conversion [4]. In Sub-Saharan Africa, around 20% of former savanna land is now cropland, with much of the remaining area used for livestock grazing [70]. The FAO predicts further encroachment of subsistence agriculture into former savanna regions due to population growth [71].

Forests ecosystems have also significantly declined, from over 500 km^2^ in 1911–60 km^2^ in 2022 (Table 3), particularly impacting endemic species (Fig 9B). This decrease varied over time and between wards. Between 1911 and 1976, many natural forest ecosystems on the western and northern slopes were converted to exotic tree plantations, while substantial forest areas on the southern slope became homegardens, and between 1976 and 2002 the Meru-Kilimanjaro forest corridor was destroyed (Fig 5). In parallel to the decreasing area of natural habitats, Fig 9A shows not only a similar negative trend over the study period, but also a similar decrease in the number of natural species at a given population density. For example, at an average population density of 1000 people/km^2^, 245 species per km^2^ were recorded in 1976, decreasing to 180 by 2003 and 115 in 2022. This decline seems linked to increasing land use intensity driven by economic development, similar to trends in natural vegetation (Fig 7). Peters et al. [68] identified land use intensity as a key factor influencing biodiversity on Kilimanjaro. Intensified land use, particularly pesticide application, significantly harms susceptible plant species, such as epiphytes in Kilimanjaro’s agroforestry systems. Chagga homegardens showed higher numbers of epiphytic mosses compared to intensively managed commercial coffee plantations [57].

Like other high mountains in tropical East Africa and Ethiopia, Kilimanjaro’s alpine zone has a high proportion of **endemic species**, with numbers decreasing at lower elevations [72]. Our dataset identified 35 endemic species, making up only 2% of the total species in Kilimanjaro’s lowlands. Fig 9B shows the declining trends of endemics in relation to population density over time.

There was also a strong negative correlation between number of **Red List species** and population density, with a clear decreasing trend over time (Fig 9C), resulting in many wards without any endangered species in 2022. IUCN Red List species (26 species in the study area) occurred only in forest and savanna habitats (Table 2), which are ecosystems with a strong decrease since 1976 (Figs 5 and 6). The relatively high number of Red List species at the highest population density of 3500 people/km^2^ is due to the presence of Rau Forest Reserve located in Moshi Municipal.

**Fig 10:**
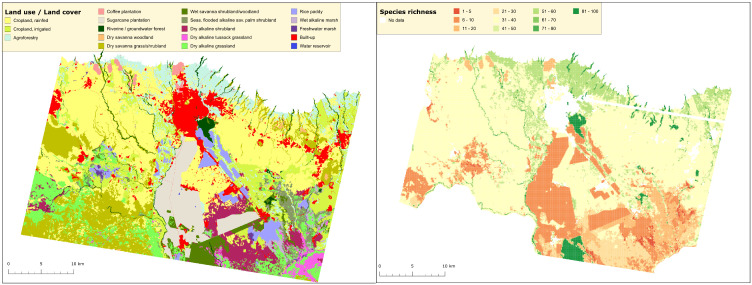
Land use and related species richness on the southern foothills of Kilimanjaro. Left: LULC map (2012) of the southern foothills of Kilimanjaro with Moshi in the centre (compiled from Hurskainen et al. 2019 [75]), right: Mean species richness at 1 ha grid. High land use intensity resulted in low diversity, whereas protected areas harboured a high biodiversity. For more details and location of the study area see Supplementary Information S1 Text and Fig 1.

**Introduced invasive** species are significant threats to biodiversity worldwide [2] and in Tanzania [73], where 67 invasive species have severe negative impacts on local ecosystems habitats and native species. Our dataset records 180 introduced species on Kilimanjaro, including cultivated plants; however, only a few, such as *Prosopis juliflora, Tecoma stans, Caesalpinia decapetala, Calliandra houstoniana, Parthenium hysterophorus* (causing allergies) and *Lantana camara*, are invasive and problematic, particularly in disturbed savanna and submontane areas, whereas *Acacia mearnsii* affects the former half-mile forest strips. A preliminary study (Hemp unpub. data) documented the spread of *Prosopis juliflora* since 1990, which now occupies 7% of the area shown in Fig 10 and is spread by livestock [74]. The other mentioned invasive species have (up to now) not significantly impacted Kilimanjaro’s ecosystems. Similarly, Jaureguiberry et al. [14] noted that, in recent decades, invasive alien species have been less important causes of global biodiversity loss, especially in Africa, compared to land-use change. In this study, neophytes are viewed not as a factor influencing biodiversity, but as a (partly negative) part of it as a consequence of land cover change.

Fig 9D shows the positive correlation between population density and neophyte numbers per km^2^. Our dataset includes 180 introduced species, representing about 9% of the total species. At 1000 people/km^2^, neophytes numbered approximately 109 in 1976, rising to 117 by 2002 and 128 in 2022, reflecting a decline in natural vegetation (Fig 4). The very weak and non-significant correlation in 2022 may be due to more open agriculture being converted into homegardens, which hold the highest number of introduced species on Kilimanjaro, with over 100 cultivated plants originating from outside Africa [53] (Table 2). Therefore, the density of introduced species peaked in rural areas rather than densely populated urban areas in 2022.

The increasing trend of neophytes may be stronger than reported, as our data cannot capture their immigration over time (see “critical remarks” in Supplementary Informations S1 Text). Increased tourism also raises the risk of introducing exotic plant species, especially in Kilimanjaro’s upper zones. One such species, *Poa annua*, a European-origin weed, is found on roadsides and pastures. Like most other neophytes on Kilimanjaro, *Poa annua* invades only anthropogenic or disturbed vegetation, occurring between about 1600 and 4000 m asl. along climbing routes or their vicinity. Its spread has paralleled the rise in climbing tourism over the past 30 years [32], though an invasion of natural vegetation types seems unlikely at present.

### The importance of nature conservation for the protection of biodiversity

The study area includes three forest reserves south of Moshi, with only Rau forest largely intact, while the others face near-total destruction, highlighting the importance of effective management in conservation to mitigate population growth’s negative effects on biodiversity. Rau Forest Reserve is increasingly attracting tourists, providing economic incentives for nature conservation in an area rich in endemic species that are found nowhere else.

The value of such reserves is clear from Fig 10, based on Hurskainen et al. [75]. In a 1300 km^2^ area on Kilimanjaro’s southern foothills (mainly within the study area, Fig 1), only 29% maintained semi-natural vegetation, with just 3% as forests in 2012. Fig 10, right panel shows species richness at 1-hectare grids; cultivated ecosystems have low species numbers, especially in high-intensity land use types like sugar cane plantations and rice fields. In contrast, Rau Forest and Namalok, a private nature reserve (see below) exhibit high species richness (84–100 species per hectare). Additionally, the critical role of riparian areas in connecting lowlands and highlands as biodiversity “blood vessels” is evident.

Another large nature reserve is Namalok. Located in the southern part of the study area (Fig 1), this reserve covers 46 km^2^ and is owned by the semi-private/state-owned sugarcane company TPC. Initial efforts to conserve this area began in 2005 and it was fenced off in 2010. Due to its remoteness, in 1976 78% was still covered by natural vegetation (riverine forests, savanna woodlands and alkaline grasslands) and by 2022 this had increased to 100%. In recent years, antelopes and zebras have been reintroduced and the reserve is now open to tourists.

## Conclusions

The lack of plant abundance data in our approach limits the application of diversity indices such as Shannon and evenness metrics. Instead, we focus on species richness, the simplest and most appropriate measure of diversity [76,77], but also on species composition. We therefore address a very significant aspect of biodiversity.

Climate change had no apparent impact on biodiversity trends in the densely populated lower regions of Kilimanjaro over the past 40 years. The primary factor negatively affecting biodiversity is land use change driven by rapid population growth and economic development that leads to increased land-use intensity replacing native biodiversity with agricultural species and ecosystems.

To our knowledge, this study is the first to link human population densities with plant species densities at a 1 km^2^ scale in a tropical region, utilizing remote sensing data alongside a comprehensive dataset of the species on the ground. This approach enabled us to analyze various plant groups of different environmental importance and not only simple total species numbers for more ecologically meaningful diversity trends. We also cleaned our dataset to avoid multiple counts of species, requiring sufficient ground data on species composition across the different vegetation types present. For this approach, extensive ecological field data are crucial, often not considered or not available. This makes it difficult to transfer this approach to other tropical regions. Furthermore, it highlights the importance of biological collections, which enable the identification and storage of collected specimens. Without the collections and taxonomists of the herbaria in Nairobi, Kew, Paris, Vienna, Oslo, Copenhagen, Berlin, Stockholm, Uppsala and Arusha, this study would not have been possible.

The declining trends in certain natural habitats would have been more pronounced if we could distinguish vegetation types at a finer scale; however, older remote sensing data limited this accuracy. For instance, wetlands – important habitats for birds on Kilimanjaro – have probably lost over 90% of their area in recent decades due to illegal water pumping and conversion to rice paddies, often abandoned after a few years due to salinization.

Most lower slopes of Kilimanjaro were already converted to anthropogenic land use by 1976, at the start of our detailed evaluation. A century ago, when Kilimanjaro’s population was below 100,000, approximately 98% of its savanna vegetation remained intact, and natural vegetation covered 90% of the lower slopes. Although biodiversity data from that time is limited, extrapolating our population density and species richness trends suggests that in 1911, nearly 700 native plant species existed per km^2^, whereas today, this number has declined to approximately 180. If we consider population density and economic prosperity as indicators of human well-being [6,78] and the area of natural habitats and species counts as indicators of ecological well-being, we can see significant gains in human well-being alongside strong declines for environmental well-being at Kilimanjaro in recent decades.

However, land degradation from poor management, such as wetland destruction and invasion by non-native species due to overgrazing former habitats of iconic animals such as elephants, giraffes and lions, threatens both the environment and long-term human well-being, despite current economic growth. For instance, asthma rates have reportedly increased on Kilimanjaro, possibly due to the replacement of permanent vegetation with fields, raising dust and allergen levels, especially during the dry seasons and exacerbated by the spread of allergenic invasive species such as *Parthenium* in disturbed areas.

On the positive side, as a combination of ecological and human well-being, the development of sustainable traditional agroforestry through the conversion of degraded land, the protection of the Rau forest compared to poorly managed reserves and the establishment of protected areas like Namalok are potential strategies to mitigate drastic changes in land cover.

Chagga homegardens stand out as a model for sustainable agricultural practices in tropical regions, showcasing land use without soil degradation or erosion for centuries. Furthermore, effective management of Kilimanjaro National Park, which provides essential ecosystem services, benefits the local population. The Namalok nature reserve, which is since 2010 protected from overgrazing by cattle, also provides space for large animals, while the local population benefits from the renewable forests as they are allowed to collect the now abundant firewood.

Kilimanjaro remains a biodiversity hotspot, reflecting both the extraordinary diversity of its flora and vegetation and the challenges posed by demographic and ecological changes, exemplifying broader issues and opportunities for other tropical regions.

## Supporting information

S1 Table Species numbers and reduction factors for all LULC-combinations. (XLSX)

S1 Text Supplementary information for “Drivers of Kilimanjaro’s decreasing biodiversity”. (DOCX)

S1 Checklist Additional information regarding the ethical, cultural, and scientific considerations specific to inclusivity in global research. (DOCX)
