# Supplementary material for: Gain and loss: Human and environmental wellbeing – drivers of Kilimanjaro’s decreasing biodiversity
Source: PLoS One. 2025 Oct 29;20(10):e0334184. doi: 10.1371/journal.pone.0334184 (PMC12571284; doi:10.1371/journal.pone.0334184)
Supplement: S1 Text — (DOCX) [file pone.0334184.s002.docx]

**Supplementary information for “Drivers of Kilimanjaro’s decreasing biodiversity”**

**Material and methods**

**Study Area**

Kilimanjaro, located in North-Eastern Tanzania, is a UNESCO World Heritage site, boasting approximately 3000 vascular plant species (Hemp, 2006b), representing one-third of Tanzania's known vascular plant species, which totals approximately 10,000 (Brenan, 1978). The study area encompasses the inhabited lower slopes and foothills below Kilimanjaro National Park (KINAPA) within five districts of Kilimanjaro Region: Moshi Municipal, Moshi Rural, Hai, Rombo, and Siha, covering 3282 km^2^ (Fig. 1).

Surrounding the mountain between 700 and 1100m is a dry and hot colline savanna zone. This zone is characterized by a heterogeneous mosaic of disturbed savanna vegetation, croplands, and built-up areas. The remaining natural vegetation faces significant pressure from human activities such as agricultural expansion and intensification, urbanization, firewood collection, brick manufacturing, and grazing (Soini 2005; Lambrechts et al. 2002; Maeda and Hurskainen 2014; Hemp and Hemp 2018).

The predominant natural vegetation comprises savanna woodlands, which have typically degraded into shrub- or grassland. Remnants of tall forest canopy are found along river courses and in areas with high groundwater tables, where (mostly heavily disturbed) lowland forests still exist. Marsh swamps are dominated by sedges, while alkaline-resistant grasses and shrubs prevail in dry vegetation on alkaline soils. Additionally, table palms dominate areas with higher groundwater on alkaline soils (Fig. 2).

Agricultural productivity in this region is limited by higher temperatures, evapotranspiration, poor soils (mainly Ferralsols and Acrisols), erratic rainfall, and reliance on irrigation (Røhr and Killingtveit, 2003; Maeda and Hurskainen, 2014). Smallholder farms cultivate extensive areas with maize, sunflower, beans, and millet. South of Moshi, large rice paddy fields and sugar cane plantations exist, while large wheat farms are present in the western part. A detailed physiographic map by Hemp et al. (2017) illustrates the different land cover and land use types (LULC) of Kilimanjaro and the study area.

Forests in the highlands within the submontane zone below KINAPA have mostly been converted into Chagga homegardens, a unique form of agroforestry prevalent on the southern and southeastern slopes. These homegardens feature four vegetation layers, maximizing land use efficiency and biodiversity, with over 500 plant species (most of them forest species) and endemic arthropods (Hemp C. 2005; Hemp 2006c).

According to the Köppen and Troll/Pfaffen climate classification system (in Müller 1983), the study area experiences a seasonal dry tropical climate influenced by the Inter-Tropical Convergence Zone. Rainfall occurs bi-modally, with rainy seasons from March to May and November. Rainfall and temperature vary with altitude and exposure to the dominant winds from the Indian Ocean (Hemp, 2006a). Mean annual temperatures range between 15 and 25°C (Appelhans et al. 2016), while annual precipitation ranges from 400 to 2800mm (Hemp, 2006c; Hemp and Hemp 2024). Southern and eastern slopes receive more rainfall due to wind exposure from the Indian Ocean compared to the northern and western slopes (Hemp, 2006a).

**Climate data**

For the temperature analysis of the last decades, we used data from the NOAA National Climatic Data Centre (NCDC) (https://www.ncdc.noaa.gov/). The temperature data are much more limited than the precipitation data, as only a few weather stations have collected long-term temperature data. The only station with useful data was Kilimanjaro Airport in the southwestern corner of the study area at 850 m a.s.l. The average daily temperature was determined from 4 to 24 measurements/day. For precipitation data, we used the published data from Otte et al. (2017). We used a generalised least squares (GLS) regression model to test the statistical significance of the long-term trends.

**Data of population and economic growth**

The scale and time frame were determined by the available census and remote sensing data: The smallest spatial unit for which census data were available was the ward level (Fig. S1) an administrative structure for one single town or portion of a bigger town. The first available Landsat image for Kilimanjaro was from 1976, and the first eligible national population census was from 1978. The other censuses were from 1988, 1998, 2012 and 2022. Due to these limitations (including the availability of cloud-free satellite imagery), we analysed the years 1976 (using the 1978 census data), 2002 and 2022. In 2007, the former Hai district was split into Siha and Hai districts. However, this had no impact on the boundaries and number of wards, which totalled 82. Between 2002 and 2022, several wards were divided and new wards were created, so that there are now 94 wards. Due to the difficulties in delineating the wards within Moshi Municipal (which is by far the smallest district with only 2 % of the study area and consists mainly of built-up areas, Fig. S1), we only considered the district as a whole and disregarded the wards of Moshi Municipal. As corresponding basis for analysing the historical maps from 1911 we used the 1913 census (Raum 1914) (see below).

We focussed on population density (number of inhabitants per km^2^). As no data on per capita income or other indicators of economic development, such as ownership of superior goods (e.g., the number of motor vehicles), were available for the entire period, we analysed house building activity as a proxy. We used a global satellite-derived built-up area statistic called GHS-BUILT-S from the Global Human Settlement Layer (GHSL) project led by the Joint Research Centre of the European Commission (Pesaresi and Politis, 2023). We analysed the built-up area over several time periods (1975, 2000 and 2020), defined as "any roofed structure erected above ground for any use", expressed in estimated square metres. Since we focussed on economic growth, we looked at both residential (RES) and non-residential (NRES) buildings from each year's strata. In parallel, we used our land cover classification to delineate built-up areas (see below).

For visualisation of these trends we digitized groups of buildings using time series of Google Earth images and the four 1:50,000 Ordnance Survey topographic sheets from the early 1990s (sheet No: Moshi 56/4, Ol Molog 42/3, Rombo 57/1, Himo 57/3).

**Land cover**

Between 1996 and 2017, over 1,600 vegetation plots were established along elevational transects on Kilimanjaro using the method of Braun-Blanquet (1964). Utilizing these vegetation data as training sites, a supervised classification (fuzzy set approach) of Landsat MSS imagery captured on 24 January 1976, and Landsat ETM imagery taken on 29 January and 21 February 2000 (source: USGS/UNEPGRID–Sioux Falls), land use/land cover (LULC) maps for 1976 and 2000 were generated (Hemp, 2005, 2006a).

To update the 2000 map to correspond to the 2002 census year, adjustments were made by visually inspecting various very high resolution (VHR) satellite imagery available at Google Earth and ESRI World Imagery platforms, correcting boundaries of agriculture/savanna and other features. Similarly, the physiographic map by Hemp et al. (2017) was updated to reflect the situation as of 2022.

Due to the lower spatial, spectral, and radiometric resolution of the Multi Spectral Scanner (MSS) on Landsat 1 satellite compared to the more advanced ETM+ and OLI sensors on later Landsat satellites, the number of LULC classes had to be reduced to match what was realistically achievable to map from the 1976 Landsat 1 image.

This led to a very rough categorisation of the main land cover and ecosystem types:

(1) agriculture (maize, sunflower and millet fields of smallholder farmers, commercial farms with wheat fields on the western and northern slopes, sugarcane plantations and rice fields south of Moshi), (2) agroforestry (including traditional chagga home gardens and commercial coffee plantations), (3) moist forests including riverine and groundwater forests and submontane ravine forests, (4) grasslands with a mosaic of anthropogenic grasslands and agricultural fields in the submontane and lower montane zone, especially on steep slopes along the major river valleys and near the national park boundary, (5) forest regeneration with vegetation recovering after previous disturbances, especially schrubland, (6) savanna with grasslands, woodlands and dry forests, (7) forest plantations with exotic tree species on the northwestern and northern slopes, 8) built-up areas and (9) water bodies (Fig. 3). We defined (8) as densely built-up areas with infrastructure, e.g. a regular network of (asphalted) roads. Since we could not delineate such areas on the Landsat image from 1978, we used the oldest available topographic maps 1.50.000 from 1982. At that time, only Moshi existed as an urban centre. For the years 2002 and 2022, we used VHR satellite images in Google Earth to delineate the urban centres with built-up areas. We also used the oldest topographic maps to delineate the boundaries of commercial estates such as the large sugar cane plantations south of Moshi (TPC) in the 1976 map as agricultural areas. The LULC maps were prepared using ArcMap 10.8.2.

Classifying LULC with sufficient accuracy is challenging using only satellite imagery (Hurskainen et al. 2019). Therefore, the 1976 map based nearly solely on satellite imagery (with the exception of built-up areas and commercial plantations) was compared with the 2002 map, which utilized a combination of image classification and visual inspection. Particularly, the analysis focused on ensuring consistency in the classification of savanna areas between 1976 and 2002, assuming that natural areas did not increase but rather decreased during this period due to population growth.

To quantify the change flows, or transitions, between LULC classes and the three time steps (1976, 2002 and 2022), we used the functions in OpenLand R library (Exavier and Zeilhofer 2021). First, the three LULC maps were cross-tabulated in order to create a contingency table, and to extract the LULC change flows between the nine classes for the entire analysed period 1976-2022, and for the three analysed time steps. Second, we visualized the change flows with a Sankey diagram, which illustrates relative transitions between the classes for the three time steps, as well as the net and gross changes with a stacked barplot.

In order to obtain an overview of the main changes in the landscape since the first census on Kilimanjaro in 1913 (Raum 1914), we also analysed historical maps from the beginning of the last century. The map of Sprigade and Moisel (1911) at a scale of 1: 300,000 provides very detailed and botanically sound descriptions of the vegetation, especially in the savanna areas of Kilimanjaro. We found more than 30 different botanical terms for the savanna vegetation, evenly distributed over the entire map. , e.g. grass and herb steppe, (dense) tree steppe, shrub steppe, salt steppe, thorn bush steppe with baobab trees, (sparse) acacia bush steppe, orchard steppe, shrub and forest vegetation. We grouped these 30+ types into a broad savanna class that also included dry savanna and most riverine forests, which we were unable to delineate. We were able to roughly delineate the lowland cultivation areas, e.g. around the Kahe river oasis, a former stopover of the caravans from the coast to the interior (Volkens 1897). There was a clear demarcation line of the montane forest that we could use, as well as delineations of the upper and lower boundaries of the Chagga homegarden cultivation zone. However, it was more difficult to delineate the large still existing submontane forest areas of this period, which was achieved with. For final delineation of the LULC types we used additional information from the map and vegetation description by Meyer (1890) together with information in Volkens (1897), Jaeger (1909) and Klute (1912).

**Biodiversity**

A detailed vegetation classification already existed for Kilimanjaro before we began our study (Hemp and Hemp 2003; Hemp, 2006b, c, 2008; Hemp et al. 2017). This classification was based on 1600 vegetation plots (releves) that were clustered according to floristic similarity using the phytosociological method of Braun-Blanquet (1964). The plots with a size between 10 (in grasslands) and 1000 m^2^ (in forests) contain structural parameters (height and vegetation cover of the herb, shrub and tree layers) in combination with a complete survey of vascular plant species of all layers (trees, shrubs, epiphytes, lianas and herbs). In seasonal environments (e.g. in savanna vegetation) they were surveyed several times, in evergreen forests mainly once.

In these earlier studies, 33 plant communities of open or cultivated habitats and 21 forest types were indicated for the entire mountain range. As our study area was limited to the lower part of this earlier study area, only a subset of these 54 land cover types occurred, represented by 772 of the 1600 releves. We combined the occurring plant communities to fit into our proposed nine classes to create species lists. For example, in class (1) agriculture we combined all open areas with open cultivation and anthropogenic plant communities with similar floristic composition (maize, rice and sugar cane plantations, ruderal vegetation on roadsides). The forest plantations with pine and cypress trees on the western and northern slopes of Kilimanjaro were also included in this class for the assessment of species richness. These forest plantations are established by local farmers growing annual agricultural crops (on Kilimanjaro mainly potatoes, carrots and cabbage) with tree seedlings in the early years, an agroforestry practice commonly referred to as the "shamba system" or "taungya system". As this class is mostly agricultural land with sparse tree cover in some areas, it is floristically similar to class (1).

The species numbers resulting from the analysis of the releves are listed in Table S1. Before these figures could be used to calculate the number of species per km^2^ (corresponding to the population density per km^2^), two further steps had to be carried out.

Firstly, we had to take into account the fact that the number of species increases with area. This species-area relationship (SAR) is one of the few very well documented patterns of species richness (Arrhenius 1921; Rosenzweig 1995). It describes the overall diversity and turnover between small and larger areas and thus enables the quantification and comparison of the spatial distribution of species (Connor and McCoy 1979; Drakare et al. 2006; Cencini et al. 2012). To this day, this model is the most commonly used and is considered the best for describing species-area calculations (Drakare et al. 2006). In a study with a subset of the 1600 releves, Hemp et al. (2021) analysed the SAR for the 13 major land use and vegetation types of Kilimanjaro. We used these calculated SAR to scale up the species counts of the nine classes to 1 km^2^. To calculate the number of species of built-up areas in cities (LULC class 8), we used the species composition of class (1), which includes ruderal vegetation types of roadsides and fallow land, but estimated that on average only 5 % of urban areas could be covered by such vegetation and reduced the number of species per km^2^ accordingly.

Secondly, there is a certain overlap of species between these classes. For example, many species of open agricultural land also occur in agroforestry systems. Based on the analysis of the releves, we calculated this overlap. In the case of the agriculture and agroforestry classes, the upscaled species counts each had to be reduced by a factor of 0.75 to avoid double counting before their species counts could be added. This overlap varies between the different classes and depends on the number of classes within a given area, in our case a ward. In one ward with all nine classes, the reduction factor was 0.51, meaning that the species numbers of each class had to be halved. Table S1 presents the species numbers and reduction factors for all combinations. Using the same approach, we calculated the number of endemic species, IUCN Red List species and neophytes from the 772 releves (Table 2). The endemic status follows a list by Gereau, Missouri Botanical Garden (pers. comm./unpublished data). We considered species in the following IUCN categories as Red List species (reviewed at https://www.iucnredlist.org/): Critically Endangered, Endangered, Vulnerable, Near Threatened. The status of neophytes was checked in FTEA (1952-2012) and Kew Plants of the World Online[(https://powo.science.kew.org/)](https://powo.science.kew.org/). In a final step, we used these reduced species numbers to calculate the final species numbers per km^2^ by relating them to the proportion of each LULC in the different areas.

We used the detailed LULC classification by Hurskainen et al. (2019) from the southern slope of Kilimanjaro to demonstrate the correlation between different LULC and species richness on a finer scale. The species richness map was created with a simple extrapolation approach: using the vegetation plot data, we first calculated the mean species richness for every LULC class. Then, we created a grid with cell size of 1 ha over the study area. Lastly, we calculated mean species richness for each grid cell, taking also into account the different LULC classes inside each grid cell. As this map was produced using a different approach and scale and represent the situation in 2012, we only used it to visualise biodiversity patterns.

**Data Analysis**

We related changes in population density at the ward level to changes in land use and vegetation cover in the respective wards. We then analysed the effects of land cover changes on biodiversity using the number of species of vascular plants as an indicator. The total number of species and the number of different plant groups (natural vegetation plants, endemic species and Red List species, neophytes) were used as indicators. To analyse trends in species numbers, we fitted linear functions using R^2^ as the measure of fit. Correlation between biodiversity data as response variable and population and land use data as explanatory variable was performed with Pearson’s correlation coefficient.

**Critical remarks**

Apart from the slightly different approach of producing the three vegetation and land cover maps we also assumed that the species pool and the species composition of the different classes did not change significantly in the period 1976-2022. This is probably true for most classes and species groups, with the exception of neophytes, which have been supplemented over time by the appearance of new species. Considering this, the observed trend would probably be more pronounced, with fewer neophytes in 1976 and a higher number in 2022. Furthermore, we had to use the 1978 census to determine the 1976 land cover classification. However, since the annual population growth in absolute numbers was lower in the 1970s than it is today, this two-year deviation should not have a significant impact on the results, especially not in the case of the deviation between the 1911 map and the 1913 census.

Comparisons of most classes based on delineations in the historical map of 1911, with the exception of the savanna, with data since 1976 are difficult. However, after combining the three classes savanna, forest and regeneration into one combined class "natural vegetation", we believe that the comparison with the analysis since 1976 is meaningful. For the year 1913, no population data were available at the ward level and no continuous climate data have been available since then. It was therefore not possible for us to analyse the changes in LULC between 1911 and 1976 with regard to driving factors such as population density on the same scale as after 1976 or climate change at all.

**References**

Appelhans T, Mwangomo E, Otte I, Detsch F, Nauss T, Hemp A (2016). Eco-meteorological characteristics of the southern slopes of Kilimanjaro, Tanzania. International Journal of Climatology 36, 3245-3258.

Arrhenius O (1921). Species and area. J Ecol 9:95–99.

Braun-Blanquet, J. (1964). Pflanzensoziologie. Wien.

Brenan JPM (1978). Some aspects of the phytogeography of tropical Africa. Annals of the Missouri Botanical Garden 65, 437-478.

Cencini M, Pigolotti S, Munoz MA (2012). What ecological factors shape species-area curves in neutral models? PLoS ONE 7:e38232.

Connor EF, McCoy ED (1979). Statistics and biology of the species-area relationship. Am Nat 113:791–833.

Drakare S, Lennon JJ, Hillebrand H (2006). The imprint of the geographical, evolutionary and ecological context on species-area relationships. Ecol Lett 9:215–227.

Exavier, R., Zeilhofer, P. (2021). OpenLand: Software for Quantitative Analysis and Visualization of Land Use and Cover Change. The R Journal 12(2): 359–371. https://doi.org/10.32614/RJ-2021-021.

FTEA (1952–2012). Flora of Tropical East Africa. Royal Botanic Gardens Kew, London.

Hemp A, Hemp J (2024). Weather or not—Global climate databases: Reliable on tropical mountains? PLoS ONE 19(3): e0299363. https://doi.org/10.1371/journal.pone.0299363.

Hemp C, Hemp A (2003): Saltatoria coenoses of high-altitude grasslands on Mt. Kilimanjaro, Tanzania (Orthoptera: Saltatoria). Ecotropica 9: 71-97.

Hemp, A. (2005). Climate change driven forest fires marginalizes the ice cap wasting on Mt. Kilimanjaro. Glob. Chang. Biol. 11 (7), 1013–1023. https://doi.org/10.1111/j. 1365-2486.2005.00968.x.

Hemp, A. (2006a). Continuum or zonation? Altitudinal gradients in the forest vegetation of Mt. Kilimanjaro. Plant Ecol. 184 (1), 27–42. https://doi.org/10.1007/s11258-005-9049-4.

Hemp, A. (2006b). Vegetation of Kilimanjaro: hidden endemics and missing bamboo. Afr. J. Ecol. 44 (3), 305–328. https://doi.org/10.1111/j.1365-2028.2006.00679.x.

Hemp, A. (2006c). The banana forests of Kilimanjaro: biodiversity and conservation of the Chagga homegardens. Biodivers. Conserv. 15 (4), 1193–1217. https://doi.org/10. 1007/s10531-004-8230-8.

Hemp, A. (2008). Introduced plants on Kilimanjaro: tourism and its impact. Plant Ecol. 197 (1), 17–29. <https://doi.org/10.1007/s11258-007-9356-z>.

Hemp, A., Hemp, C. (2018). Broken bridges. The isolation of Kilimanjaro’s ecosystem. Glob. Chang. Biol. 24 (8), 3499–3507. <https://doi.org/10.1111/gcb.14078>.

Hemp, A., Oleson, E., Buchroithner, M.F. (2017). Kilimanjaro. Physiographic Map With Landuse and Vegetation, Scale 1:100,000. ARGE, München, Germany.

Hemp, A., Oleson, E., Buchroithner, M.F. (2017). Kilimanjaro. Physiographic Map With Landuse and Vegetation, Scale 1:100,000. ARGE, München, Germany.

Hemp, C. (2005). The Chagga Home Gardens: Relict Areas for Endemic Saltatoria Species (Insecta: Orthoptera) on Mt. Kilimanjaro. Biodiversity and Conservation 125: 203-10.

Hurskainen P, Adhikaria H, Siljandera M, Pellikka PKE, Hemp A (2019). Auxiliary datasets improve accuracy of object-based land use/land cover classification in heterogeneous savannah landscapes. Remote Sensing of Environment 233, 111354. (DOI: 10.1016/j.rse.2019.111354)

Jaeger, F. (1909). Forschungen in den Hochregionen des Kilimandscharo. Mitteilungen aus den Deutschen Schutzgebieten 22: 113-146; 161-196.

Klute, F. (1920). Ergebnisse der Forschungen am Kilimandscharo 1912. Berlin. 136 pp.

Lambrechts C, Woodley B, Hemp A, Hemp C, Nnyiti P (2002). Aerial survey of the threats to Mt. Kilimanjaro forests. United Nations Development Programme (UNDP), Dar es Salaam, Tanzania. (URL: http://wedocs.unep.org/bitstream/handle/20.500.11822/9563/Mt_Kilimanjaro_Report_Aerial_survey_2001.pdf?sequence=3&isAllowed=y)

Maeda, E.E., Hurskainen, P. (2014). Spatiotemporal characterization of land surface temperature in Mount Kilimanjaro using satellite data. Theor. Appl. Climatol. 118 (3), 497–509. <https://doi.org/10.1007/s00704-013-1082-y>.

Meyer, H. (1890). Ostafrikanische Gletscherfahrten. Forschungsreisen im Kilimandscharo-Gebiet. – Leipzig: Duncker & Humblot, 376 pp.

Müller, M.J. (1983). Handbuch ausgewählter Klimastationen der Erde, 3. Edition. Forschungsstelle Bodenerosion der Universität Trier Mertesdorf, Ruwertal (Trier).

Otte I, Detsch F, Mwangomo E, Hemp A, Appelhans T, Nauss T (2016). Multidecadal trends and interannual variability of rainfall as observed from five lowland stations at Mt. Kilimanjaro, Tanzania. Journal of Hydrometeorology 18, 349-361.

Pesaresi M. and Politis P. (2023). GHS-BUILT-S R2023A - GHS built-up surface grid, derived from Sentinel2 composite and Landsat, multitemporal (1975-2030), European Commission, Joint Research Centre (JRC) PID: http://data.europa.eu/89h/9f06f36f-4b11-47ec-abb0-4f8b7b1d72ea, doi:10.2905/9F06F36F-4B11-47EC-ABB0-4F8B7B1D72EA.

Raum, J. (1914). Statistisches aus dem Dschaggalande. Evangelisch-lutherisches Missionsblatt 1914: 124-7.

Røhr, P.C., Killingtveit, Å. (2003). Rainfall distribution on the slopes of Mt Kilimanjaro. Hydrol. Sci. J. 48 (1), 65–77. https://doi.org/10.1623/hysj.48.1.65.43483.

Rosenzweig ML (1995). Species diversity in space and time. Cambridge University Press, Cambridge

Shongwe ME, van Oldenborgh GJ, van den Hurk B, van Aalst M (2011). Projected changes in mean and extreme precipitation in Africa under global warming. Part II: East Africa. Journal of Climate 24, 3718-3733.

Soini E (2005). Land use change patterns and livelihood dynamics on the slopes of Mt. Kilimanjaro. Agricultural Systems 85, 306-323.

Sprigade, P. & Moisel, M. (1911). Karte von Deutsch-Ostafrika. B5. Kilimandscharo. Reimer, Berlin.

Volkens G. (1897). Der Kilimandscharo: Darstellung der allgemeineren Ergebnisse eines fünfzehnmonatigen Aufenthalts im Dschaggalande. Berlin: Reimer.
